# Prevalence and comorbidity of mental disorders among young adults with a history of residential youth care – a two-wave longitudinal study of stability and change

**DOI:** 10.1007/s00406-025-02007-x

**Published:** 2025-04-27

**Authors:** Hanne Klæboe Greger, Nanna Sønnichsen Kayed, Stine Lehmann, Thomas Jozefiak, Stian Lydersen, Lars Wichstrøm, Katrine Kveli Fjukstad

**Affiliations:** 1 Department of Mental Health, Norwegian University of Science and Technology, Trondheim, Norway; 2 Department of Mental Healthcare – Emergency and Children, St. Olavs Hospital, Trondheim, Norway; 3 Department of Clinical Psychology, Faculty of Psychology, University of Bergen, Bergen, Norway; 4 Department of Psychology, Norwegian University of Science and Technology, Trondheim, Norway; 5 Department of Psychiatry, Nord-Trøndelag Hospital Trust, Levanger Hospital, Levanger, Norway

**Keywords:** Residential youth care, Developmental psychopathology, Mental health, Child welfare

## Abstract

Among youth in residential youth care (RYC) there is a high load of experienced adversities and a high prevalence of mental disorders. Less is known about the stability and change of mental disorders and whether individual or contextual factors that are protective of adult mental disorders could be identified. The current study is a 10-year follow-up of young adults with a history of RYC in Norway. At baseline (T1), 76% met criteria of a mental disorder. At follow-up (T2), diagnostic psychiatric interviews were conducted by phone by experienced doctors and psychologists. A response rate of 52% was achieved (n = 157). Criteria for at least one present mental disorder were fulfilled by 77.6%. The most prevalent single disorders were PTSD (42.1%), agoraphobia (33.3%), social phobia (27.9%), major depressive episode (23.1%), specific phobia (21.1%), and ADHD (19.2%). Most participants had more than one mental disorder. Among those with a diagnosis at T1, completing high school was the only factor found to be associated with no diagnosis at T2 (p = 0.019), whereas no statistically significant association was found with sex, age at first placement and received aftercare. We found a very high prevalence of mental disorders and a high rate of comorbidity comparable to the prevalence at baseline. This shows high stability of psychopathology from adolescence into adulthood. Ensuring access to high quality mental health services for young adults with a history of living in RYC is important. Prevention and early intervention, such as aid to complete education, should be prioritized for young people with high risk of developing mental disorders. Response rate, sample size and selection bias are discussed as possible limitations to the study.

**Supplementary Information:**

The online version contains supplementary material available at 10.1007/s00406-025-02007-x.

## Introduction

Children and adolescents living in Residential Youth Care (RYC) have been exposed to a high burden of childhood adversities such as maltreatment [22], placement instability [24, 50], and disrupted education [35]. These circumstances may increase the risk of mental health problems and impaired functioning [21, 34]. In Norway, placement in RYC is only considered if less intrusive measures, like placement in foster families, are deemed insufficient [1]. In 2023, 889 children and adolescents lived in RYC in Norway [46]. These young people have a high prevalence and comorbidity of mental disorders, with rates as high as 76% [26]. They also report poor quality of life (QoL) and a high prevalence of maltreatment experiences [21, 52]. This high prevalence is in line with other international studies on adolescents in RYC [9, 10, 27, 41]. A wide range of services are often necessary to help young adults with a history of RYC to settle into adult life, including the need for evidence-based and individually tailored mental health treatment. To aid this, knowledge is needed on the prevalence and continuity of mental disorders in young adulthood for this group, including individual risk and protective factors of mental disorders.

The age of onset of most mental disorders coincides with substantial biological, psychological and social changes that occur in the transition from childhood to adolescence and early adulthood. The transition phase from childhood to adulthood can entail difficulties in adapting to independence while handling socioeconomic adversities like unemployment and poor living conditions, as well as ongoing mental health disorders. A meta-study of general population samples found that among those with a mental disorder, the first onset was before the age of 14 in one-third, before the age of 18 in half, and before the age of 25 in two-thirds of the cases [45]. Mental disorders in childhood and adolescence increase the risk of mental disorders in adulthood [11, 36]. Both in the general population and in high-risk populations, mental disorders in adolescence persist into adulthood, causing impaired functioning and QoL [34, 44].

In a report from the Norwegian Institute of Public Health, the prevalence of mental disorders among adults in Norway is reported to be 16–22% [48]. The most prevalent disorders are anxiety (15%) and depression (10 %). The prevalence of mental disorders is substantially higher among youth living in RYC than in the general population [42]. Whether this high prevalence continues into adulthood or if it wanes remains relatively unknown. A recent meta-study showed a high prevalence rate of mental disorders among adults with a child welfare care history (30 %) and in adults with a juvenile justice care history (45 %), compared to the prevalence rate in the general adult population (18 %) [42]. Another study reported a rate of 70% of any mental disorder 10 years after placement by the Child Welfare Services (CWS), suggesting that on a group level, the increased risk continues. There are also indications that there is continuity on the individual level, with high stability of mental disorders from adolescence into adulthood among those placed by the CWS [41].

Seker et al. found a high risk of continuous psychopathology among adolescents transitioning out of RYC; however, knowledge about individual and contextual factors that can improve mental health outcomes is highly needed. In Norway, aftercare is provided by the CWS to adolescents 18–25 years as they age out of care, to give necessary support in the transition to independent adulthood. Whether received aftercare improves mental health outcomes in adulthood is unknown. The prevalence of childhood maltreatment is very high among adolescents in RYC and is a risk factor for mental disorders later in life [10]. As out-of-home placements by the CWS, to a large degree, is an intervention to protect children from abuse and neglect, the age of first placement could be of importance regarding total exposure to childhood maltreatment. However, it is possible that those placed at a very early age have been exposed to more severe adversities than those placed in adolescence, and hence even out the protective effect of early placement. Lastly, length of education has been shown to be associated with a lower prevalence of mental disorders later in life and with reduced adult mortality [6, 29]. Based on international studies, adolescents aging out of RYC are at increased risk of mental disorders as adults. Received aftercare, age at first placement in out-of-home care, and high school completion are possible predictors of adult mental health outcomes in individuals with a history of living in RYC institutions and could help to guide service providers in developing preventive interventions.

In this prospective 10-year follow-up study, we investigate the long-term outcome for young adults with a history of living in RYC. More specifically, we examine 1) the point-prevalence and comorbidity of mental disorders at follow-up. 2) stability and change in mental disorders across two time points spanning 10 years. 3) whether individual (sex, high school completion) or contextual (age at first placement, use of aftercare) factors predict the absence of mental disorder at T2 among individuals with a mental disorder at T1.

## Methods

### Procedure and sample

#### The baseline study (T1)

During the period of June 2011 to July 2014, all Norwegian RYC institutions with children and adolescents aged 12–23 years were invited to participate in the study with a few exclusions (unaccompanied minors without resident permit, acute crisis placement, insufficient proficiency in Norwegian language) [26]. A total of 86 out of 94 institutions accepted the invitation, and 400 out of 601 eligible adolescents participated (67% response rate, 57.5% females, age range 12–20 years, mean age = 16.8 years). At T1, we conducted an attrition analysis based on the Child Behavior Checklist [2] completed by the main contact at the institution for participants, and for 141 anonymous non-participants. There were more males among non-participants, and they had higher problem-scores on five out of eight subscales, but with small effect sizes [26]. Written informed consent was obtained, and if the participant was under 16 years, consent from the guardian was also obtained. All 400 participants from baseline consented to be contacted for follow-up. For details of the baseline study, see Jozefiak et al. 2016 [25].

#### The follow-up study (T2)

At T2, 10 years after T1, all participants at T1 were invited to participate in a follow-up study. Personal identification numbers were coupled with an official registry containing phone numbers and e-mail addresses of residents in Norway. The young adult was invited to participate by completing an online survey and a subsequent telephone interview. Attempts to contact eligible participants through SMS and phone calls were repeated until contact was obtained, within the limit of three attempts. If no response was obtained, an information letter with an invitation to participate was sent via both e-mail and postal mail. The recruitment period for the VINGO study started Jan 5^th^ 2021 and ended April 17^th^ 2023. Participants received a gift card (500 NOK = 44 EUR) after they completed the telephone interview. All participants were also included in a lottery for a larger value gift-card (10 000 NOK = 875 EUR).

We established contact with 302 individuals. We found that 10 had deceased, and for 18 individuals, no valid contact information could be found. A total of 65 refused participation following contact, and 80 individuals did not complete the consent form or survey, despite reminders. As a result, a total of 157 young adults participated, corresponding to a 52% response rate of eligible participants. Attrition analyses showed that compared to non-participants at T2, the VINGO participants were more often females (68.2% vs 50.8%) and had a higher mean number of psychiatric disorders at baseline (2.6 (SD 2.6) vs 2.0 (SD 2.3)). More specifically, they had higher baseline prevalence of depression, anxiety, and conduct disorder. They also had a higher proportion of reported suicide attempts, and problematic substance use at baseline. Sample characteristics are shown in Table 1. For details of the recruitment and measures in the follow-up study, see Greger et al 2024 [23].

**Table 1:** Sample characteristics of the 157 participants at follow-up

	n	%
Female	107	68.2
Age (mean (SD))	25.4 (1.6)	
Birth country Norway	145	92.4
Own children (n = 156)	45	28.8
Highest degree of completed education (n = 154)		
1–12 years	99	64.3
13 years (high school)	23	14.9
Certificate of apprenticeship (vocational training)	22	14.3
1 or more years of higher education	10	6.5
Employed	63	40.1
Unemployed	21	13.4
In education	23	14.6
Disability benefits (100%)	34	21.7
Dropout of education you wanted to complete	111	70.7
Personal economy		
Bad/very bad	70	44.6
Okay	56	35.7
Good/very good	31	19.7

### User involvement

The design of the VINGO study was created in cooperation with user representatives with lived experience from CWS or child and adolescent mental health services. A user representative took part in project meetings where design and method were discussed, focus group interviews were conducted to ensure relevant questions for the survey and interview in the study.

### Measures

#### Sociodemographic information

Information on age, sex, education, employment status, marital status and economy were collected in the online survey. Personal economy was measured by subjective report of how the individual considered their own economy, with five alternatives (very bad, bad, okay, good, very good).

#### Mental disorders

At T2, a semi-structured psychiatric assessment using the M.I.N.I. international neuropsychiatric interview (M.I.N.I plus) [43] was conducted through a telephone interview. The M.I.N.I. plus sections included in the current study were depression, dysthymia, suicidality, mania, panic disorder, agoraphobia, social phobia, specific phobia, obsessive-compulsive disorder, alcohol and drug use/dependency, psychosis, eating disorder, generalized anxiety disorder, somatoform disorder, somatoform pain disorder and attention deficit hyperactivity disorder (ADHD). In addition, post-traumatic stress disorder (PTSD) was assessed by using the Posttraumatic Stress Disorder Checklist for DSM-5 (Norwegian version; TRAPS) [7, 19]. TRAPS is a two-part questionnaire primarily used as a screening instrument, but it was administered as a part of the diagnostic interview in this study. The first part is based on Stressful Life Events Screening Questionnaire [19] and is an assessment of 15 different potentially traumatic life events, such as accidents, natural disasters, terror attacks, war, physical violence, and sexual abuse. The second part consists of 20 questions about posttraumatic stress symptoms, compliant with the DSM-5 Criteria for PTSD. To secure an optimal diagnostic evaluation, the M.I.N.I. plus and the TRAPS were administered by clinical psychologists and medical doctors with experience from mental health services and diagnostic competence. A total of nine interviewers (seven psychologists and two doctors) participated in digital meetings for discussion and training before the data collection. The interviewers made diagnostic conclusions based on the participants’ answers to the M.I.N.I plus and the TRAPS interview.

Prior to analyses, the first and last authors (HKG and KKF) selected the diagnoses available from the M.I.N.I. plus considered most clinically relevant (shown in Table 2). The following diagnostic group variables were computed: “Any anxiety disorder” includes agoraphobia, panic disorder, social phobia, specific phobia, generalized anxiety disorder (GAD) (including GAD due to medical condition and substance-related GAD), obsessive-compulsive disorder (OCD) (including OCD due to medical condition and substance-related OCD), somatoform pain disorder, and somatization disorder. “Any depressive disorder” includes major depressive episode, major depressive disorder, dysthymic disorder, depression due to medical condition, and substance-related mood disorder. “Psychotic disorder” includes psychotic disorder (not otherwise specified), psychosis due to medical condition, substance-related psychosis, schizophrenia, brief psychotic episode, and delusional disorder. “Somatoform pain disorder” includes somatoform pain disorder with psychological factors, and somatoform pain disorder with psychological factors and general medical condition. To calculate the mean number of disorders for the participants, a sum score was computed, ranging from 0 to 10, including the following ten categories of diagnoses: any depressive disorder, any anxiety disorder, bipolar disorder, psychotic disorder, alcohol abuse or dependency, substance abuse or dependency, anorexia nervosa, bulimia nervosa, PTSD and ADHD. A total list of diagnoses from the M.I.N.I. plus interview is presented in the supplementary material (S3).

**Table 2:** Point-prevalence of mental disorders by sex, n %

Disorder	Total n = 147	Female n = 102	Male n = 45	Difference estimate (95% CI)	p
Major depressive episode	34 (23.1)	23 (22.5)	11 (24.4)	1.9 (− 17.8 to 11.7)	0.82
Dysthymic disorder	20 (13.6)	16 (15.7)	4 (8.9)	6.8 (− 6.4 to 16.7)	0.29
Hypomanic episode	3 (2.0)	0	3 (6.7)	**6.7 (**− **17.9 to −1.0)**	**0.009**
Manic episode	2 (1.4)	1 (1.0)	1 (2.2)	1.2 (− 10.6 to 3.5)	0.72
Bipolar disorder	6 (4.1)	2 (2.0)	4 (8.9)	**6.9 (−** **18.9 to 0.4)**	**0.049**
Panic disorder	22 (15.0)	17 (16.7)	5 (11.1)	5.6 (− 8.2 to 16.1)	0.40
Agoraphobia	49 (33.3)	38 (37.3)	11 (24.4)	12.9 (− 3.9 to 26.9)	0.14
Panic anxiety with agoraphobia	25 (17.0)	18 (17.6)	7 (15.6)	2.0 (− 12.5 to 13.7)	0.77
Social phobia	41 (27.9)	32 (31.4)	9 (20.0)	11.4 (− 4.7 to 24.6)	0.16
Specific phobia	31 (21.1)	24 (23.5)	7 (15.6)	7.9 (− 7.1 to 20.0)	0.30
Obsessive compulsive disorder (OCD)	21 (14.3)	17 (16.7)	4 (8.9)	7.8 (− 5.5 to 17.8)	0.23
Generalized anxiety disorder (GAD)*	14 (9.6)	13 (12.7)	1 (2.3)	10.4 (− 0.4 to 18.5)	0.05
Somatization disorder*	16 (11.0)	15 (14.7)	1 (2.3)	**12.4 (1.4 to 20.8)**	**0.028**
Somatoform pain disorder*	10 (6.8)	7 (6.9)	3 (6.8)	0.1 (− 11.9 to 8.0)	1.00
PTSD**	61 (42.1)	48 (47.5)	13 (29.5)	**18.0 (0.5 to 32.9)**	**0.045**
Anorexia nervosa*	0				
Bulimia nervosa*	8 (5.5)	6 (5.9)	2 (4.5)	1.4 (− 9.7 to 8.5)	0.77
ADHD*	28 (19.2)	18 (17.6)	10 (22.7)	5.1 (− 20.6 to 8.0)	0.49
Psychotic disorder*	5 (3.4)	5 (4.9)	0	4.9 (− 3.6 to 11.0)	0.14
Alcohol dependency*	15 (10.3)	10 (9.8)	5 (11.4)	1.6 (− 14.9 to 8.2)	0.80
Alcohol abuse*	12 (8.2)	10 (9.8)	2 (4.5)	5.3 (− 6.2 to 13.3)	0.33
Drug/substance dependency*	17 (11.6)	7 (6.9)	10 (22.7)	**15.8 (−** **30.6 to −4.0)**	**0.006**
Drug/substance abuse*	10 (6.8)	4 (3.9)	6 (13.6)	**9.7 (−23.0 to −0.5)**	**0.034**
Any depressive disorder	46 (31.3)	33 (32.4)	13 (28.9)	3.5 (− 13.2 to 18.2)	0.69
Any anxiety*	90 (61.6)	70 (68.6)	20 (45.5)	**23.1 (5.8 to 39.2)**	**0.009**
Any disorder	114 (77.6)	81 (79.4)	33 (73.3)	6.1 (− 7.8 to 21.9)	0.43
Number of disorders**					
No disorder	31 (21.4)	20 (19.8)	11 (25.0)		
1 disorder	34 (23.4)	23 (22.8)	11 (25.0)		
2 disorders	33 (22.8)	22 (21.8)	11 (25.0)		
3 disorders	25 (17.2)	21 (20.8)	4 (9.1)		
4 + disorders	22 (15.1)	15 (14.9)	7 (15.8)		
Mean number of disorders (SD)**	1.9 (1.6)	2.0 (1.6)	1.8 (1.7)		0.79

^*^n = 146 (female n = 102, male n = 44)

^**^n = 145 (female n = 101, male n = 44)

At T1, we used the CAPA interview for diagnostic assessment. The CAPA is a semi-structured psychiatric interview designed to gather information from children and adolescents. It contains required and optional questions to ensure that the interviewer gets the information required for diagnostic conclusions according to the DSM-IV of a broad spectrum of psychiatric disorders [5]. Adolescents were not considered fully reliable reporters of symptoms of ADHD, autism spectrum disorder, and reactive attachment disorder. Information concerning these diagnoses was therefore collected from their main contact at the institution using the parent and the preschool age version of the CAPA interview, and the Asperger Syndrome Diagnostic Interview [17].

### Statistics

Inter-rater reliability for diagnostic conclusion was quantified as positive and negative agreement, as recommended by de Vet [49]. The positive and negative agreement have interpretations similar to sensitivity and specificity.

Differences between proportions in independent samples (Tables 2, 3 and 5) were analyzed using methods which can handle small counts such as is the case in some of our comparisons. We used the Newcombe Hybrid Score Confidence interval for differences between proportions, as recommended by [14, 16]. P-values for differences between proportions were computed using the unconditional z-pooled test as recommended by [14] and [30]. These analyses were conducted using the R package “contingency tables” [18]. A significance level of 0.05 was chosen, and 95% confidence intervals (CI) are reported where relevant (in bold).

**Table 3:** Comorbid diagnosis pairs by sex, n (%)

Comorbid diagnosis pairs	Total n = 146	Female n = 102	Male n = 44	Difference estimate (95% CI)	p
Dep+Anx	37(25.3)	29 (28.4)	8 (18.2)	10.2 (− 5.6 to 23.1)	0.21
Dep+ADHD	9 (6.2)	6 (5.9)	3 (6.8)	0.9 (− 12.8 to 6.8)	0.84
Dep+PTSD*	31 (21.4)	24 (23.8)	7 (15.9)	7.9 (− 7.4 to 20.0)	0.31
Dep+Psy/bip	4 (2.7)	4 (3.9)	0	3.9 (− 4.5 to 9.7)	0.23
Dep+Alc	8 (5.5)	5 (4.9)	3(6.8)	1.9 (− 13.7 to 5.6)	0.76
Dep+Sub	6 (4.1)	2 (2.0)	4 (9.0)	**7.0 (**− **19.3 to** **0.2)**	**0.046**
Anx+ADHD	22 (15.1)	15 (14.7)	7(15.9)	1.2 (− 15.8 to 10.2)	0.88
Anx+PTSD*	49 (33.8)	40 (39.6)	9 (20.5)	**19.1 (2.5 to** **32.6)**	**0.026**
Anx+Psy/bip	8 (5.5)	6 (5.9)	2 (4.5)	1.4 (− 9.7 to 8.5)	0.77
Anx+Alc	12 (8.2)	8 (7.8)	4 (9.0)	1.2 (− 13.9 to 7.6)	0.82
Anx+Sub	12 (8.2)	6 (5.9)	6 (13.6)	7.7 (− 21.2 to 1.9)	0.13
ADHD+PTSD*	17 (11.7)	12 (11.9)	5 (11.4)	0.5 (− 13.0 to 10.6)	0.99
ADHD+Psy/bip	6 (4.1)	4(3.9)	2 (4.5)	0.6 (− 11.5 to 6.0)	0.90
ADHD+Alc	8 (5.5)	5(4.9)	3(6.8)	1.7 (− 13.7 to 5.6)	0.76
ADHD+Sub	9 (6.2)	4(3.9)	5 (11.4)	7.5 (− 20.3 to 1.2)	0.09
PTSD+Psy/bip*	5 (3.4)	5 (5.0)	0	5.0 (− 3.6 to 11.1)	0.14
PTSD+Alc*	11 (7.6)	8 (7.9)	3(6.8)	1.1 (− 10.9 to 9.4)	0.85
PTSD+Sub*	8 (5.5)	4 (4.0)	4(9.0)	5.0 (− 17.4 to 2.9)	0.24
Psy/bip+Alc	4 (2.7)	3 (2.9)	1 (2.3)	0.6 (− 9.1 to 6.3)	0.84
Psy/bip+Sub	2 (1.4)	1 (1.0)	1 (2.3)	1.3(− 10.9 to 3.5)	0.72
Alc+Sub	5 (3.4)	1 (1.0)	4 (9.0)	**8.0 (− 20.2 to − 1.1)**	**0.013**

*Dep* any depressive disorder, *Anx* any anxiety disorder, *Psy/bip* any psychotic or bipolar disorder, *Alc* alcohol dependency, *Sub* Drug or substance dependency

^*^n=145 (female n = 101, male n = 44)

The prevalence of psychopatology at T1 and T2 (Table 4) was compared using the asymptotic McNemar test as recommended by [15].

**Table 4:** Mental disorders across two time-points, n (% of total)

	Any diagnosis T2		
Any diagnosis T1	Yes	No	Total
Yes	79 (55.2)	26 (18.2)	105 (73.4)
No	33 (23.1)	5 (3.5)	38 (26.6)
Total	112 (78.3)	31 (21.7)	143 (100)

### Ethics

The baseline study was approved by the Regional Ethical Committee for Medical and Health Research, Mid-Norway (REK) (2010/1965/REK Midt), and the VINGO study was approved by the Norwegian Agency for Shared Services in Education and Research (Sikt ref: 790618) and by REK (502016/REK Midt). At T1, written informed consent was obtained from all participants. For youth under 16 years, a written informed consent from the guardian was obtained. At T2, written informed consent was gathered before enrolment in the follow-up study, including consent to merge data from the national registries mentioned above.

## Results

### Sample characteristics

The mean age of the participants was 25.4 years, and 68.2 % were female. The majority (64.3 %) stated that 12 years of elementary school was the highest degree of completed education, and 40.1 % were employed. See Table 1 for more details.

### Inter-rater reliability

Inter-rater agreement of diagnostic conclusion was studied based on tape-recorded interviews of the M.I.N.I. plus interview and the TRAPS interview. We planned to rescore 30 interviews. However, two interviews were excluded due to lack of registration from rater 1, and an interrupted interview by the participant. Hence, a total of 28 interviews were rescored. In total, 7 of the 9 interviewers took part in the rescoring. The design is shown in supplementary table S1.

Selected diagnoses from the M.I.N.I. plus and the TRAPS interviews were studied. Where several sub-categories of a diagnosis were present, or where a diagnosis was either previous and/or present, group variables were constructed. A total of 29 diagnosis variables were studied, including the variable “any diagnosis” both on T1 and T2 data. Details are shown in supplementary table S2. To sum up, negative agreement was high, ranging from 0.80 to 1.00 for all diagnoses except depression, where negative agreement was 0.63. Negative agreement for any ongoing diagnosis was 0.80. As expected, due to some of the diagnoses being low-frequent, positive agreement varied more: 0.67–1.00 for 15 diagnoses, and 0.40–0.62 for 6 diagnoses. Positive agreement for schizophrenia, bipolar disorder II, somatoform pain disorder, and delusional disorder were 0. For the diagnosis anorexia nervosa all were diagnosed negative by both raters, the negative agreement is 1 and positive agreement cannot be computed. Positive agreement for any ongoing diagnosis was 0.96. Positive (0.85) and negative (0.81) agreement based on T1 data was calculated for any diagnosis and found to be high.

### Prevalence of mental disorders

The point prevalence of mental disorders stratified by sex is presented in Table 2. Criteria for at least one present mental disorder were fulfilled by 77.6%. The most prevalent single disorders were PTSD, agoraphobia, social phobia, major depressive episode, specific phobia, and ADHD. Of notice, the prevalence of bipolar disorder was 4.1%, and of psychotic disorder was 3.4%. PTSD and somatization disorders were more common among females, while bipolar disorder, drug dependency, and drug abuse were more common among males.

### Comorbidity of mental disorders

**Fig. 1:**
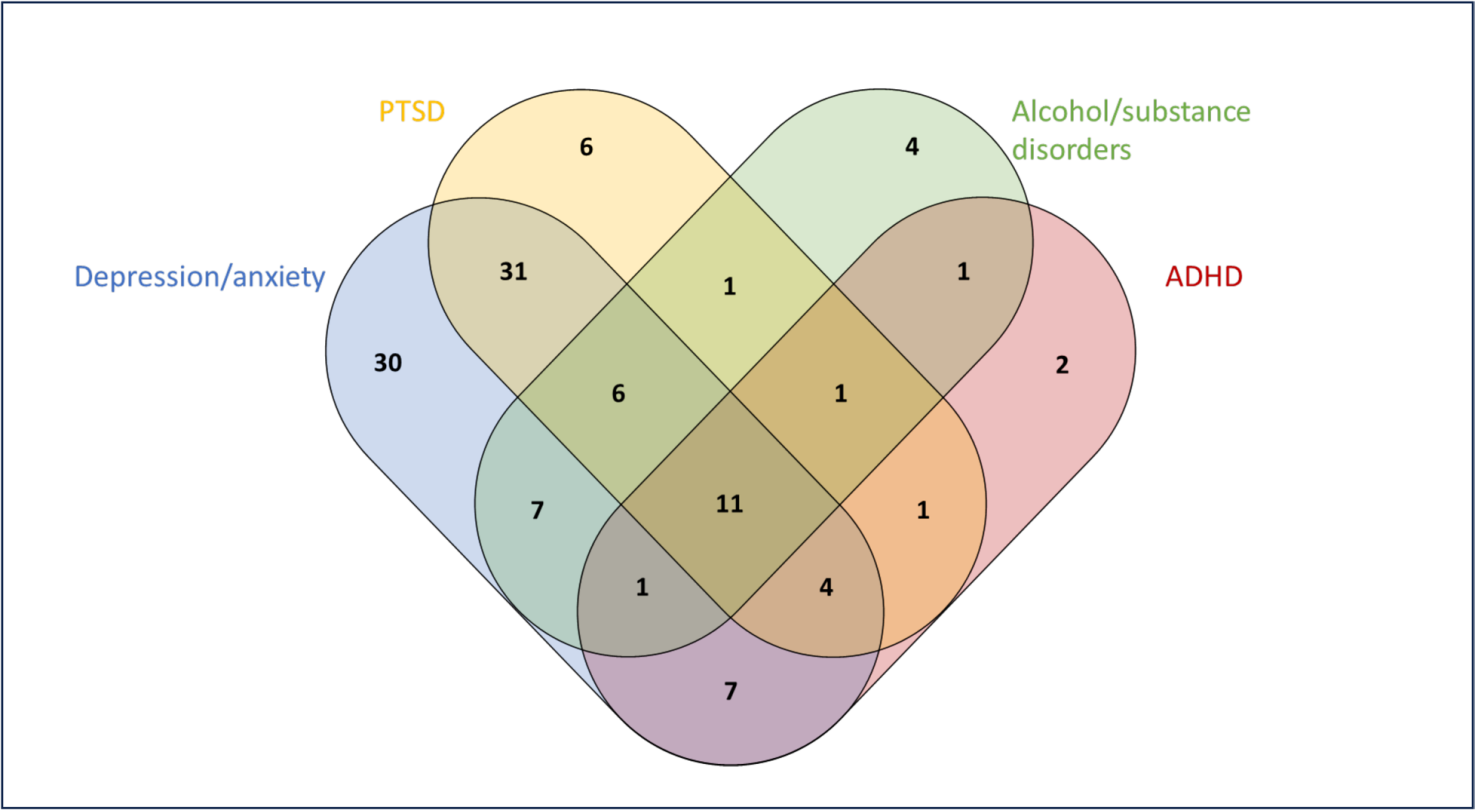
Numbers of participants with overlapping diagnostic categories. Total number of participants in each category: Depression/anxiety (n = 97), PTSD (n = 61), Alcohol/substance disorders (n = 32), ADHD (n = 28). Participants with no or other disorders: n =32

Table 2 shows that a high proportion of the individuals fulfilled the criteria for 2, 3, or even 4 or more diagnoses. The mean number of mental disorders (1.9) also points to a high rate of comorbidity. Table 3 shows the prevalence of common diagnosis pairs. One out of three had comorbid anxiety and PTSD, one out of four had comorbid depression and anxiety, and more than one out of five had comorbid depression and PTSD. Comorbid anxiety and PTSD were more common among females. Comorbid substance dependency and depression, and comorbid substance dependency and alcohol dependency were more common among males. Figure 1 shows the overlap of four selected diagnostic categories.

### Stability and predictors of mental disorders

Table 4 shows that very few of the participants were found to have no diagnoses at T1 and T2 (n = 5), and the largest group of participants was given a diagnosis both on T1 and T2. The prevalence of diagnoses at T1 and T2 was 73.4% and 78.3%, but the difference was not statistically significant (McNemar’s test, p = 0.36). Among the 105 participants with a diagnosis at T1, completing high school was the only factor found to be associated with no diagnosis at T2 (p = 0.019), whereas no statistically significant association was found with sex, age at first placement and received aftercare (Table 5).

**Table 5:** Predictors and diagnostic status at T2 among the 105 participants who fulfilled criteria for at least one psychiatric diagnosis at T1

	Diagnosis at T2		
Predictor	Yes	No	p
Sex n (%)			0.97
Male	24 (75.0)	8 (25.0)	
Female	55 (75.3)	18 (24.7)	
Completed high school or vocational training n (%)			**0.019**
Yes	20 (60.6)	13 (39.4)	
No	59 (81.9)	13 (18.1)	
Received aftercare^1^ n (%)			0.94
Yes	56 (75.7)	18 (24.3)	
No	21 (75.0)	7 (25.0)	
Age at first placement^1^ (mean years)	12.4	12.9	0.64^2^

1: by the CWS

2: Two sample t-test

## Discussion

The current 10-year follow-up study shows that young adults with a history of living in RYC have a very high overall prevalence of mental disorders (77.6%), and a high rate of comorbidity. The rates are much higher than in the Norwegian general population where the prevalence is 16–22% [48], but comparable to the prevalence at baseline, were 76.3% filled diagnostic criteria of a mental disorder [26]. Overall, our results show high stability of psychopathology from adolescence into adulthood. Completed high school education was identified as a potential protective factor against adult psychopathology among individuals with mental disorders in adolescence.

Emotional disorders (anxiety and depression) and PTSD were the most prevalent of the disorders. These disorders are associated with childhood maltreatment [12, 20, 32]. At T1, 71% of the adolescents in the study reported exposure to physical violence, sexual abuse or witnessing violence [22]. In addition, most of the residents in RYC will have experiences of emotional and/or physical neglect [33]. Therefore, a high prevalence of anxiety, depression and PTSD is to be expected. However, we also found that psychotic disorders and bipolar disorders were prevalent in the current population. These severe mental disorders are often associated with loss of function, high degree of morbidity and mortality, and the need for long-term health- and social services. Both genetic and environmental factors, such as traumatic childhood experiences, are found to be risk factors for the development of psychotic disorders and bipolar disorders [37, 38, 40]. Even though the interaction between genetic and environmental factors is not fully understood, the individuals in RYC might be in double jeopardy where heredity and adversity coincide and create even more challenging conditions for childhood development. Of note, whereas our findings of high rates of PTSD may be explained by high exposure to potentially traumatic events during childhood, we cannot rule out that the symptomatology identified in this follow-up study refers to more recent experiences with victimization. Due to their high exposure to child maltreatment, these young adults are at particular risk of revictimization also after placement [8, 51]. To prevent ongoing detrimental exposure to interpersonal violence after placement, RYC institutions need to build competency in early identification and prevention of violence from peers and intimate partners against residents in their care.

Females had higher rates of PTSD and somatization disorders compared to males, whereas males were overrepresented in bipolar disorder, drug abuse and -dependency. Our findings are in line with previous research which show a 2:1 ratio of PTSD in females compared to males [13]. A higher prevalence of drug abuse and dependency in males is well documented, but the gender gap is narrowing [31].

Even though point-prevalence rates at follow-up are similar to that of baseline, this does not imply a stable prevalence of single mental disorders in this population. Comorbid mental disorders are common in clinical populations, and other studies have found that all mental disorders are risk factors for all other mental disorders [39]. Our results correspond well with the results reported by Seker et al. [41]. In their 10-year follow-up of Swiss adolescents in RYC, they found an overall prevalence of 83% of mental disorders. Our findings strengthen the notion that young adults with a background in child protection services are at particularly high-risk, and in need of continued access to services specialized in interventions tailored to meet long-term consequences of childhood adversities such as maltreatment, placement instability, and disrupted education. The skewed distribution of mental disorders between young adults with a history of living in RYC institutions and the general population necessitates a response that reduces these inequities [28]. Kirkbridge et al. present recommendations to handle the social determinants of mental health, which, among other aspects, include both universal primary prevention of mental illnesses, which may involve family interventions, and handling individual social determinants like maltreatment and neglect [28].

Among participants with a mental disorder at T1, we found that completed education at high school level was the only factor that predicted the absence of mental disorders at T2. One reason for this result could be that participants with a mental disorder might have a very low function level and, therefore, not be able to engage in education and drop out of school because of their impairment. This is supported by an Australian prospective study of more than 5000 children, which found that abuse and neglect were associated with cognitive delay and poor educational outcomes in adolescence and early adulthood [47]. Moreover, we cannot preclude that those being able to complete a high school education had additional resources not accounted for, such as higher IQ, better executive functioning, being more conscientious, or living in RYC with routines and structures facilitating school attendance, and a supportive social network, which also protects against future mental disorders. However, our results could also indicate that interventions that improve school presence and completion may be an important preventive measure for later mental disorders in this high-risk population. Adolescents in RYC are at risk of poor educational outcomes, but knowledge of effective school-focused interventions for adolescents in RYC is very limited [3]. Our results underline the need for further research on effective interventions that increase educational outcomes for this population.

Neither sex, age at first placement, nor received aftercare was associated with present mental disorder at T2. As early age at first placement can be explained by a mix of very severe adversity (that would pose as a potent risk factor for mental disorder in adulthood) and proper protective actions by the CWS (that would pose as a protective factor), this could be the cause of lacking associations in the analysis. Aftercare is supposed to be a service tailored to the individuals needs and was therefore hypothesized as a possible protective factor. The lack of association could be an indicator of poor quality of these services. However, received aftercare was reported by the participant themselves, and the lack of association could also be caused by the participant not remembering receiving aftercare services.

### Strengths and limitations

The current study is one of very few follow-up studies of adolescents living in RYC. It therefore represents a unique contribution to the knowledge of a high-risk population transitioning to adulthood. The high involvement of user representatives throughout the VINGO project is a strength. User representatives have been involved in all phases of the study, from planning, designing, and creating costume-made questions for the survey and interview through focus-group interviews. We have available diagnostic information from two time points, gathered by personal interviews, to increase diagnostic validity. Interrater agreement for conclusion on any diagnosis was high in both our datasets. This indicates that the stability of psychopathology found in the study population among those participants who filled diagnostic criteria at T1 is not due to variation in rater conclusions. There were only 5 participants that did not have any mental disorder at both time-points. This could be an indicator of selection bias in the sample where individuals with a high burden of psychiatric disorders agreed to participate at T2 in a larger extent than healthy individuals.

At T2, medical doctors and clinical psychologists performed the interviews. As in-person diagnostic evaluation was not possible due to limitations in the financial resources of the study, telephone interviews were chosen as assessment method. Therefore, the diagnostic conclusions can be considered as close to gold-standard as possible in such research. However, we have to take into account that the high prevalence of ADHD and substance abuse, and the somewhat low prevalence of bipolar disorder could be due to differential diagnostic challenges. Emotional dysregulation, impulsivity and psychomotor agitation can represent symptoms of bipolar disorder, ADHD and substance use disorders, as these may overlap. This represents an uncertainty in our material, as some of the symptoms might end up having a different etiology or represent an undiscovered comorbidity [4]. In addition, not all diagnoses were included in both interviews. For example, attachment disorders and autism spectrum disorders were assessed at T1 but not T2, and somatization disorder was assessed at T2 but not at T1. Therefore, the group variables constructed at T2 are not directly comparable to those from the T1 dataset. However, the overall prevalence of mental disorders and the prevalence of the specific disorders are comparable, as both diagnostic interviews are based on DSM-IV criteria.

Despite extensive effort, we recruited only 157 out of the 400 T1 participants for the VINGO study. We established contact with 302 individuals; 65 refused participation following contact, and 80 individuals did not complete the consent form or survey despite reminders. This resulted in a response rate of 52% of the eligible participants. The recruitment procedure revealed a high mortality rate (10/400) in this sample. From the T1 data, we had information on a very high prevalence of adolescent mental disorders, substance use, and suicidal ideation. Contradictory to what could be expected, the T2 participants had more mental disorders than non-participants at T1. This reduces the risk of underestimating the results but could also be an indicator of selection bias. Given this population with a very high load of burdens, we consider the response rate satisfactory. However, the resulting sample size prevented some subgroup analyses (i.e., predictors for psychopathology among those without mental disorders at T1). Although we identify associations in the current study, this does not equal causal effects.

## Conclusion

Young adults who have aged out of RYC have a very high prevalence of mental disorders and a high rate of comorbid disorders, including severe disorders such as psychosis and bipolar disorder. Ensuring access to high-quality mental health services and care for these individuals is of great importance. Preventive measures that can improve long-term outcomes should be implemented early on. Our study indicates that completing education at the high school level might be an important preventive factor that should be given priority by social and health authorities. Further research on effective interventions to improve educational outcomes and mental health is highly needed. Focusing on prevention and early intervention for young people with an especially high risk of developing mental disorders, such as young adults with a history of living in RYC, should be prioritized.

## Supplementary Information

Below is the link to the electronic supplementary material.

Supplementary file1 (DOCX 30 KB)
