# Supplementary material for: Prevalence and comorbidity of mental disorders among young adults with a history of residential youth care – a two-wave longitudinal study of stability and change
Source: Eur Arch Psychiatry Clin Neurosci. 2025 Apr 27;276(1):39–49. doi: 10.1007/s00406-025-02007-x (PMC12904880; doi:10.1007/s00406-025-02007-x)
Supplement: Supplementary file 1 — Supplementary file1 (DOCX 30 KB) [file 406_2025_2007_MOESM1_ESM.docx]

Table S1

Study design of inter-rater agreement of diagnostic conclusion

|  |  | Rater 2 | | | | | | | | | |
| --- | --- | --- | --- | --- | --- | --- | --- | --- | --- | --- | --- |
| Rater 1 |  | A | B | C | D | E | F | G | H | I | Sum |
|  | A | 0 | 0 | 1 | 1 | 0 | 0 | 0 | 1 | 0 | 3 |
|  | B | 1 | 0 | 1 | 0 | 0 | 1 | 0 | 0 | 0 | 3 |
|  | C | 0 | 0 | 0 | 0 | 1 | 1 | 0 | 1 | 0 | 3 |
|  | D | 1 | 0 | 0 | 0 | 0 | 1 | 1 | 1 | 0 | 4 |
|  | E | 1 | 0 | 0 | 0 | 0 | 1 | 1 | 0 | 0 | 3 |
|  | F | 0 | 0 | 1 | 0 | 1 | 0 | 1 | 1 | 0 | 3 |
|  | G | 1 | 0 | 1 | 0 | 1 | 0 | 0 | 0 | 0 | 3 |
|  | H | 1 | 0 | 0 | 0 | 1 | 0 | 1 | 0 | 0 | 3 |
|  | I | 0 | 0 | 0 | 0 | 1 | 1 | 1 | 0 | 0 | 3 |
|  |  |  |  |  |  |  |  |  |  |  |  |
|  | Sum | 5 | 0 | 4 | 1 | 4 | 5 | 5 | 4 | 0 | 28 |

Table S2

Interrater agreement

**ADHD**

|  | Rater 2  No | Yes | Positive agreement | Negative agreement |
| --- | --- | --- | --- | --- |
| Rater 1 No | 20 | 2 | 0,86 | 0,95 |
| Yes | 0 | 6 |  |  |

**Agoraphobia**

|  | Rater 2  No | Yes | Positive agreement | Negative agreement |
| --- | --- | --- | --- | --- |
| Rater 1 No | 14 | 2 | 0,88 | 0,90 |
| Yes | 1 | 11 |  |  |

**GAD**

|  | Rater 2  No | Yes | Positive agreement | Negative agreement |
| --- | --- | --- | --- | --- |
| Rater 1 No | 24 | 1 | 0,40 | 0,94 |
| Yes | 2 | 1 |  |  |

**OCD**

|  | Rater 2  No | Yes | Positive agreement | Negative agreement |
| --- | --- | --- | --- | --- |
| Rater 1 No | 26 | 0 | 1,00 | 1,00 |
| Yes | 0 | 2 |  |  |

**Social phobia**

|  | Rater 2  No | Yes | Positive agreement | Negative agreement |
| --- | --- | --- | --- | --- |
| Rater 1 No | 18 | 2 | 0,82 | 0,92 |
| Yes | 1 | 7 |  |  |

**Specific phobia**

|  | Rater 2  No | Yes | Positive agreement | Negative agreement |
| --- | --- | --- | --- | --- |
| Rater 1 No | 20 | 2 | 0,67 | 0,91 |
| Yes | 2 | 4 |  |  |

**Panic anxiety**

|  | Rater 2  No | Yes | Positive agreement | Negative agreement |
| --- | --- | --- | --- | --- |
| Rater 1 No | 10 | 3 | 0,84 | 0,80 |
| Yes | 2 | 13 |  |  |

**PTSD**

|  | Rater 2  No | Yes | Positive agreement | Negative agreement |
| --- | --- | --- | --- | --- |
| Rater 1 No | 14 | 1 | 0,78 | 0,85 |
| Yes | 4 | 9 |  |  |

**Depression**

|  | Rater 2  No | Yes | Positive agreement | Negative agreement |
| --- | --- | --- | --- | --- |
| Rater 1 No | 5 | 5 | 0,85 | 0,63 |
| Yes | 1 | 17 |  |  |

**Dysthymic disorder**

|  | Rater 2  No | Yes | Positive agreement | Negative agreement |
| --- | --- | --- | --- | --- |
| Rater 1 No | 19 | 3 | 0,62 | 0,88 |
| Yes | 2 | 4 |  |  |

**Hypomania**

|  | Rater 2  No | Yes | Positive agreement | Negative agreement |
| --- | --- | --- | --- | --- |
| Rater 1 No | 26 | 1 | 0,67 | 0,98 |
| Yes | 0 | 1 |  |  |

**Mania**

|  | Rater 2  No | Yes | Positive agreement | Negative agreement |
| --- | --- | --- | --- | --- |
| Rater 1 No | 22 | 3 | 0,67 | 0,94 |
| Yes | 0 | 3 |  |  |

**Bipolar I**

|  | Rater 2  No | Yes | Positive agreement | Negative agreement |
| --- | --- | --- | --- | --- |
| Rater 1 No | 24 | 3 | 0,40 | 0,94 |
| Yes | 0 | 1 |  |  |

**Bipolar II**

|  | Rater 2  No | Yes | Positive agreement | Negative agreement |
| --- | --- | --- | --- | --- |
| Rater 1 No | 25 | 2 | 0,00 | 0,94 |
| Yes | 1 | 0 |  |  |

**Psychotic disorder**

|  | Rater 2  No | Yes | Positive agreement | Negative agreement |
| --- | --- | --- | --- | --- |
| Rater 1 No | 17 | 5 | 0,43 | 0,81 |
| Yes | 3 | 3 |  |  |

**Schizophrenia**

|  | Rater 2  No | Yes | Positive agreement | Negative agreement |
| --- | --- | --- | --- | --- |
| Rater 1 No | 27 | 1 | 0,00 | 0,98 |
| Yes | 0 | 0 |  |  |

**Delusional disorder**

|  | Rater 2  No | Yes | Positive agreement | Negative agreement |
| --- | --- | --- | --- | --- |
| Rater 1 No | 25 | 1 | 0,00 | 0,94 |
| Yes | 2 | 0 |  |  |

**Alcohol dependency**

|  | Rater 2  No | Yes | Positive agreement | Negative agreement |
| --- | --- | --- | --- | --- |
| Rater 1 No | 25 | 0 | 0,80 | 0,98 |
| Yes | 1 | 2 |  |  |

**Alcohol abuse**

|  | Rater 2  No | Yes | Positive agreement | Negative agreement |
| --- | --- | --- | --- | --- |
| Rater 1 No | 24 | 1 | 0,86 | 0,98 |
| Yes | 0 | 3 |  |  |

**Drug dependency**

|  | Rater 2  No | Yes | Positive agreement | Negative agreement |
| --- | --- | --- | --- | --- |
| Rater 1 No | 17 | 3 | 0,78 | 0,89 |
| Yes | 1 | 7 |  |  |

**Drug abuse**

|  | Rater 2  No | Yes | Positive agreement | Negative agreement |
| --- | --- | --- | --- | --- |
| Rater 1 No | 25 | 2 | 0,50 | 0,96 |
| Yes | 0 | 1 |  |  |

**Substance-related depressive disorder**

|  | Rater 2  No | Yes | Positive agreement | Negative agreement |
| --- | --- | --- | --- | --- |
| Rater 1 No | 22 | 2 | 0,50 | 0,92 |
| Yes | 2 | 2 |  |  |

**Substance-related psychotic disorder**

|  | Rater 2  No | Yes | Positive agreement | Negative agreement |
| --- | --- | --- | --- | --- |
| Rater 1 No | 18 | 4 | 0,67 | 0,88 |
| Yes | 1 | 5 |  |  |

**Somatization disorder**

|  | Rater 2  No | Yes | Positive agreement | Negative agreement |
| --- | --- | --- | --- | --- |
| Rater 1 No | 23 | 1 | 0,57 | 0,94 |
| Yes | 2 | 2 |  |  |

**Somatoform pain disorder**

|  | Rater 2  No | Yes | Positive agreement | Negative agreement |
| --- | --- | --- | --- | --- |
| Rater 1 No | 27 | 1 | 0,00 | 0,98 |
| Yes | 0 | 0 |  |  |

**Bulimia nervosa**

|  | Rater 2  No | Yes | Positive agreement | Negative agreement |
| --- | --- | --- | --- | --- |
| Rater 1 No | 25 | 1 | 0,80 | 0,98 |
| Yes | 0 | 2 |  |  |

**Anorexia nervosa**

|  | Rater 2  No | Yes | Positive agreement | Negative agreement |
| --- | --- | --- | --- | --- |
| Rater 1 No | 28 | 0 | * | 1,00 |
| Yes | 0 | 0 |  |  |

**Any (ongoing) diagnosis (T2)**

|  | Rater 2  No | Yes | Positive agreement | Negative agreement |
| --- | --- | --- | --- | --- |
| Rater 1 No | 4 | 0 | 0,96 | 0,80 |
| Yes | 2 | 22 |  |  |

**Any diagnosis (T1)**

|  | Rater 2  No | Yes | Positive agreement | Negative agreement |
| --- | --- | --- | --- | --- |
| Rater 1 No | 15 | 3 | 0,85 | 0,81 |
| Yes | 4 | 20 |  |  |

Table S3

Prevalence of mental disorders at T2

| **Disorder** | **n= 147** | **%** |
| --- | --- | --- |
| Psychotic disorder NOS (ongoing) | 5/146 | 3.4 |
| Psychotic disorder NOS (previous) | 17/146 | 11.6 |
| Psychotic disorder due to gen medical condition (ongoing) | 0 |  |
| Psychotic disorder due to gen medical condition (previous) | 1/146 | 0.7 |
| Substance-related psychotic disorder (ongoing) | 3/146 | 2.1 |
| Substance-related psychotic disorder (previous) | 27/146 | 18.5 |
| Schizophrenia (ongoing) | 1/146 | 0.7 |
| Schizophrenia (previous) | 0 |  |
| Brief psychotic disorder (ongoing) | 2/146 | 1.4 |
| Brief psychotic disorder (previous) | 15/146 | 10.3 |
| Delusional disorder (ongoing) | 1/146 | 0.7 |
| Delusional disorder (previous) | 4/146 | 2.7 |
| Major depressive disorder (ongoing) | 16 | 10.9 |
| Major depressive disorder (previous) | 49 | 33.3 |
| Bipolar I (ongoing) | 3 | 2.1 |
| Bipolar I (lifetime) | 8 | 5.5 |
| Bipolar II (ongoing) | 3 | 2.1 |
| Bipolar II (lifetime) | 5 | 3.4 |
| Depr/mania/hypomania subtype – rapid cycling | 25 | 17.0 |
| Depr/mania subtype mixed episode | 14 | 9.5 |
| Depr/mania/hypomania subtype – annual pattern | 10 | 6.8 |
| Depr/mania/hypomania subtype – full remission between episodes | 20 | 13.6 |
| Panic disorder lifetime | 53 | 36.1 |
| Panic disorder w limited symptoms lifetime | 22 | 15.0 |
| Panic disorder ongoing | 22 | 15.0 |
| Anxiety w panic attacks due to ongoing medical condition (ongoing) | 2 | 1.4 |
| Substance-related anxiety disorder w panic attacks (ongoing) | 3 | 2.0 |
| Agoraphobia (lifetime) | 63 | 42.9 |
| Agoraphobia (ongoing) | 49 | 33.3 |
| Panic anxiety wo agoraphobia (ongoing) | 8 | 5.4 |
| Panic anx w agoraphobia (lifetime) | 25 | 17.0 |
| Agoraphobia wo history of panic anxiety (ongoing) | 14 | 9.5 |
| Agoraphobia wo ongoing panic anx, but history of panic anx (ongoing) | 10 | 6.8 |
| Agoraphobia (ongoing) wo history of panic anx w limited symptoms | 7 | 4.8 |
| Social phobia (ongoing) | 41 | 27.9 |
| Specific phobia (ongoing) | 31 | 21.1 |
| OCD (ongoing) | 20 | 13.6 |
| OCD due to somatic | 0 |  |
| Substance-related OCD (ongoing) | 2 | 1.4 |
| Alcohol dependency (ongoing) | 15/146 | 10.3 |
| Alcohol abuse (ongoing) | 12/146 | 8.2 |
| Drug/substance dependency lifetime | 52/146 | 35.6 |
| Drug/substance dependency (ongoing) | 17/146 | 11.6 |
| Drug/substance abuse (ongoing) | 10/146 | 6.8 |
| Major depressive disorder w psychotic symptoms | 13/62 | 21.0 |
| Bipolar I disorder w psychotic symptoms | 4/12 | 33.3 |
| Anorexia nervosa | 0 |  |
| Bulimia nervosa (ongoing) | 8/146 | 5.5 |
| GAD (ongoing) | 14/146 | 9.6 |
| GAD due to somatic disorder (ongoing) | 1/146 | 0.7 |
| Substance-related GAD (ongoing) | 2/146 | 1.4 |
| Somatization disorder lifetime | 13/146 | 8.9 |
| Somatization disorder (ongoing) | 16/146 | 11.0 |
| Somatoform pain disorder w psychological factors (ongoing) | 7/146 | 4.8 |
| Somatoform pain disorder w psychological factors and general medical condition (ongoing) | 5/146 | 3.4 |
| ADHD | 28/146 | 19.2 |
| PTSD (ongoing) | 61/145 | 42.1 |
| Major depressive episode (ongoing) | 34 | 23.1 |
| Major depressive episode (lifetime) | 95 | 64.6 |
| Affective disorder (depr) due to gen medical condition (ongoing) | 0 |  |
| Affective disorder (depr) due to gen medical condition (lifetime) | 2 | 1.4 |
| Substance-related mood disorder (ongoing) | 1 | 0.7 |
| Substance-related mood disorder (lifetime) | 15 | 10.2 |
| Dysthymic disorder (ongoing) | 20 | 13.6 |
| Dysthymic disorder (lifetime) | 22 | 15.0 |
| Hypomanic episode (ongoing) | 3 | 2.0 |
| Hypomanic episode (lifetime) | 24 | 16.3 |
| Manic episode (ongoing) | 2 | 1.4 |
| Manic episode (lifetime) | 17 | 11.6 |
| Manic (hypomanic) episode due to gen medical condition (ongoing) | 0 |  |
| Manic (hypomanic) episode due to gen medical condition (lifetime) | 1 | 0.7 |
| Substance-related manic (hypomanic) episode (ongoing) | 0 |  |
| Substance-related manic (hypomanic) episode (lifetime) | 13 | 8.8 |

NOS: not otherwise specified; gen: general; w: with; wo: without; depr: depression; anx: anxiety
